# Dynamics of gas exchange and heart rate signal entropy in standard cardiopulmonary exercise testing during critical periods of growth and development

**DOI:** 10.14814/phy2.70034

**Published:** 2024-09-11

**Authors:** Zachary Blanks, Donald E. Brown, Dan M. Cooper, Shlomit Radom Aizik, Ronen Bar‐Yoseph

**Affiliations:** ^1^ School of Data Science, University of Virginia Charlottesville Virginia USA; ^2^ Institute for Clinical and Translational Science University of California Irvine California USA; ^3^ Department of Pediatrics, Pediatric Exercise and Genomics Research Center University of California Irvine California USA; ^4^ Pediatric Pulmonary Institute, Ruth Rappaport Children's Hospital, Rambam Health Care Campus Haifa Israel

**Keywords:** cardiopulmonary exercise testing, informatics in exercise testing, pediatric exercise, pubertal differences, sample entropy

## Abstract

Standard cardiopulmonary exercise testing (CPET) produces a rich dataset but its current analysis is often limited to a few derived variables such as maximal or peak oxygen uptake (V̇O_2_). We tested whether breath‐by‐breath CPET data could be used to determine sample entropy (SampEn) in 81 healthy children and adolescents (age 7–18 years old, equal sex distribution). To overcome challenges of the relatively small time‐series CPET data size and its nonstationarity, we developed a Python algorithm for short‐duration physiological signals. Comparing pre‐ and post‐ventilatory threshold (VT_1_) CPET phases, we found: (1) SampEn decreased by 9.46% for V̇O_2_ and 5.01% for V̇CO_2_ (*p* < 0.05), in the younger, early‐pubertal participants; and (2) HR SampEn fell substantially by 70.8% in the younger and 77.5% in the older participants (*p* < 0.001). Across all ages, females exhibited greater HR SampEn than males during both pre‐ and post VT_1_ CPET phases by 14.10% and 23.79%, respectively, *p* < 0.01. In females, late‐pubertal had 17.6% lower HR SampEn compared to early‐pubertal participants (*p* < 0.05). Breath‐by‐breath gas exchange and HR data from CPET are amenable to SampEn analysis that leads to novel insight into physiological responses to work intensity, and sex and maturational effects.

## 1 INTRODUCTION

The goal of this research was to apply data analytic approaches to heart rate (HR), respiratory rate (RR), and gas exchange (oxygen uptake, V̇O_2_; carbon dioxide output, V̇CO_2_; ventilation, V̇E) responses to exercise to test hypotheses focused on the effect of work intensity, maturational status, and biological sex on signal entropy and variability. Progressive cardiopulmonary exercise testing (CPET) is the mainstay of laboratory assessments of cardiorespiratory‐metabolic fitness (CRMF) in a variety of diseases and conditions (Blanks, Brown, Cooper, et al., 2024). As the intensity of work performed by the testing participant increases using either a treadmill (TM) or cycle ergometer (CE), gas exchange can be measured breath‐by‐breath providing a rich data source for subsequent analysis. Both maximal and near maximal variables (e.g., peak V̇O_2_) as well as submaximal variables (e.g., ΔV̇E/ΔV̇CO_2_) can elucidate fundamental mechanisms and abnormalities in the integrated physiologic response to exercise (Wu et al., 2016). Consequently, a single, relatively brief, albeit highly effort dependent, visit to the laboratory for children, adolescents, and adults, can provide useful and actionable insights for both researchers and clinicians across a wide spectrum of human health and disease (Harber et al., 2024; Smith et al., 2022).

Although breath‐by‐breath gas exchange technologies have been available for four decades (Kiefer et al., 2021), the predominant analytic approach to CPET data has been, with exception of HR in which extensive analysis of variability has been performed (Pincus et al., 2000), to overlook the complexity (often and perhaps mistakenly referred to as noise) of time series gas exchange variables in an effort to produce straightforward outcomes useful for both clinical and research purposes. Peak V̇O_2_, for example, typically requires averaging or smoothing of breath‐to‐breath values (Lai et al., 2023) over a domain of exercise estimated to be at high intensity. Approaches to submaximal CPET usually involves linear modeling with the estimated calculation of a single variable, such as a slope (e.g., ΔV̇O_2_/ΔHR). Dynamic responses, such as kinetics of gas exchange during on‐ or off‐transients in response to exercise, are increasingly seen as useful indicators of cardiorespiratory‐metabolic impairment. To produce a parsimonious set of parameters that accurately describe gas exchange kinetics such as the system gain and time constant, fairly complex curve fitting models often with combinations of higher order exponential and linear equations, are used and require data reduction, extensive smoothing, and a wide range of assumptions (Solís‐Montufar et al., 2020).

In this research we examined the utility of information science analytics such as entropy, originally developed for the digital information transmitted through noisy channels (Berry et al., 2022), in the context of physiological responses to exercise. The study focused on the most common laboratory CPET protocols in which work intensity increases progressively until the limit of the participant's tolerance is reached, typically after 10–12 min. We recently demonstrated the potential usefulness of this approach in a different exercise protocol that we had specifically developed for children and adolescents, the multiple brief exercise bouts (MBEB). MBEB consists of ten 2‐min constant work rate CE exercise periods interspersed with 1 min of rest. The MBEB study provided new insights into the effect of work intensity, biological sex, and pubertal status on physiological exercise responses (Blanks, Brown, Cooper, et al., 2024).

Information entropy analysis of typical, progressive exercise CPET, however, poses a number of methodological challenges. Information entropy analysis was originally designed for very large data sets. Even the relatively small 500–1000 distinct observations in MBEB were greater than the usual ~200 distinct observations in progressive CPET. Moreover, the concept of stationarity, an assumption of earlier applications of informational entropy analytics that the mean, variance and autocorrelation structure of a time‐series data set does not change over time, is clearly not applicable to progressive exercise CPET. In a recent study, we addressed this challenge by developing an algorithm specifically suited for time‐series data sets of relatively short duration (Blanks & Brown, 2024). In the present study, we present an approach to evaluating the variability and complexity of physiological signals obtained from early‐ and late‐pubertal children using typical progressive exercise CPET and outline key insights regarding the development of the exercise response derived from this analysis during a critical period of growth and development.

## 2 MATERIALS AND METHODS

### 2.1 Sample entropy overview

Sample entropy (SampEn) serves as a measure for assessing the predictability of time series data by evaluating the likelihood of “templates” remaining within a specified tolerance level.

Let $x\in {\mathbb{R}}^{N}$ be a time series signal consisting of N real‐valued observations. A template is defined as a vector of length m (referred to as the “embedding dimension”) containing consecutive elements from x. Specifically, the i‐th template is an m‐dimensional vector, $x_{m}^{\left(i\right)}=\left(x_{i},x_{i+1},\ldots ,x_{i+m-1}\right).$ The comparison between templates involves assessing whether their distance is less than a predefined similarity radius r>0. A match, indicating regularity in the time series data, occurs when:

$${\left\|x_{m}^{\left(i\right)}-x_{m}^{\left(j\right)}\right\|}_{\infty }=\max_{k=1,\ldots m}\left|x_{m}^{\left(i\right)}\left(k\right)-x_{m}^{\left(j\right)}\left(k\right)\right|\leq r,$$

where $x_{m}^{\left(i\right)}\left(k\right)$ is the k‐th element of the template. This template‐matching process is repeated for all combinations of reference and comparison templates (excluding self‐checks). Mathematically, this quantity is denoted as $B^{m}\left(r\right)$, representing the probability that the time series data will remain within a distance r for m points, calculated as:

(1) $$B^{m}\left(r\right)=\frac{1}{Z\left(N,m\right)}\sum_{i=1}^{N-m}\sum_{j=1;i\neq j}^{N-m-1}\Theta \left[{\left\|x_{m}^{\left(i\right)}-x_{m}^{\left(j\right)}\right\|}_{\infty }\leq r\right].$$

Here, $\Theta \left[\cdot \right]$ corresponds to the Heaviside function, taking the value 1 if the condition holds and 0 otherwise. $Z\left(N,m\right)$ is a normalization constant ensuring $B^{m}\left(r\right)$ lies between zero and one. This calculation process is repeated for templates of size m+1, and the probability that the data will remain within a distance r r for m+1 points is computed as $A^{m}\left(r\right)$, given by:

(2) $$\begin{array}{c} A^{m}\left(r\right)=\frac{1}{Z\left(N,m\right)}\sum_{i=1}^{N-m}\sum_{j=1;i\neq j}^{N-m-1}\Theta \left[{\left\|x_{m+1}^{\left(i\right)}-x_{m+1}^{\left(j\right)}\right\|}_{\infty }\leq r\right]. \end{array}$$

The SampEn of x for parameters $\left(m,r\right)$ is then calculated as:

(3) $$\begin{array}{c} \text{SampEn}\left(x,m,r\right)=-\log\frac{A^{m}\left(r\right)}{B^{m}\left(r\right)}. \end{array}$$

If $B^{m}\left(r\right)=0$, SampEn is undefined. If $B^{m}\left(r\right)>0$, but $A^{m}\left(r\right)=0$, then $\text{SampEn}\left(x,m,r\right)=\infty$. For a detailed discussion of SampEn's mathematical foundations, interested readers can refer to Delgado‐Bonal & Marshak. (2019) and Richman & Moorman. (2000).

### 2.2 Automated ventilatory threshold detection

We studied a participant's entropy before and after reaching their ventilatory threshold (VT_1_) during a maximum‐effort CPET. The VT_1_ is a well‐established and physiologically significant point in exercise physiology, representing a transition in a participant's metabolic response (Rossiter, 2021). We hypothesized that a participant's entropy would decrease following this exercise phase, indicating a more regular and predictable physiological pattern. By investigating these changes, we aimed to gain insights into the dynamics and complexity of the participant's physiological response during the CPET.

To study this hypothesized change in entropy, we employed an automated VT_1_ detection protocol developed by Kim et al. (2021). The authors demonstrated the statistical equivalence of their approach to trained observers using a nine‐panel plot and employed two approaches: the excess CO_2_ (ExCO_2_) method and the V‐slope method (Beaver et al., 1986).

The VT_1_ detection problem can be formulated as an optimization task, where we seek to partition a time‐series signal $x\in {\mathbb{R}}^{N}$, where N denotes the number of observations in the signal and ℝ is the real numbers, into two segments, $x_{0}$ and $x_{1}$, to minimize a combination of log‐variance terms. This is expressed by the following optimization problem:

(4) $$\begin{array}{c} \min_{k\in \mathbb{N}}\;\left(k-1\right)\log V\left(x_{0}\right)+\left(N-k+1\right)\log V\left(x_{1}\right).\; \end{array}$$

Here, $x_{0}$ represents the initial segment of the signal with k data points, while $x_{1}$ comprises the remaining data points from k+1 to N. The objective is to find the optimal time index k∈ℕ where ℕ is the set of natural numbers that defines the ventilatory threshold. The empirical variance of a signal is denoted by $V\left(\cdot \right)$.

To estimate the VT_1_ for a participant, we calculated the optimal values by solving Equation (Smith et al., 2022) using both the V‐slope and ExCO_2_ methods. We employed dynamic programming and utilized an open‐source implementation provided by Truong, Oudre, and Vayatis (Truong et al., 2020) to compute the optimal partitioning of the time‐series signal. By averaging the results obtained from both methods, we aimed to improve the reliability of the estimated VT_1_, reducing potential bias and variability associated with a single approach (Ekkekakis et al., 2008).

### 2.3 Constructing weakly stationary signals

To ensure a valid entropy analysis of the CPET data, it is essential to work with weakly stationary signals (Richman & Moorman, 2000). Empirical studies have emphasized the importance of signal stationarity for accurate entropy analysis (Chatain et al., 2020). However, in the context of a maximum effort CPET, the original signal set is likely to have a non‐constant mean due to the increasing work rate throughout the test, making it non‐stationary. Therefore, we applied a differencing algorithm to transform the signals into a weakly stationary form before conducting downstream analyses.

Let x be a signal from the CPET signal set, then the differenced signal, $\tilde{x}$, is calculated via:

(5) $$\begin{array}{c} {\tilde{x}}_{t}=x_{t}-x_{t-1},t=2,\ldots ,N, \end{array}$$

where N is the total number of observations in x. We standardized all the signals in the updated signal set to have zero mean and unit variance, and checked if the resulting signals were weakly stationary at significance level α=0.05 using the Augmented Dickey‐Fuller (ADF) test and corrected for multiple testing errors using the Holm‐Sidak method (Dickey & Fuller, 1979; Holm, 1979; Šidák, 1967).

**FIGURE 1:**
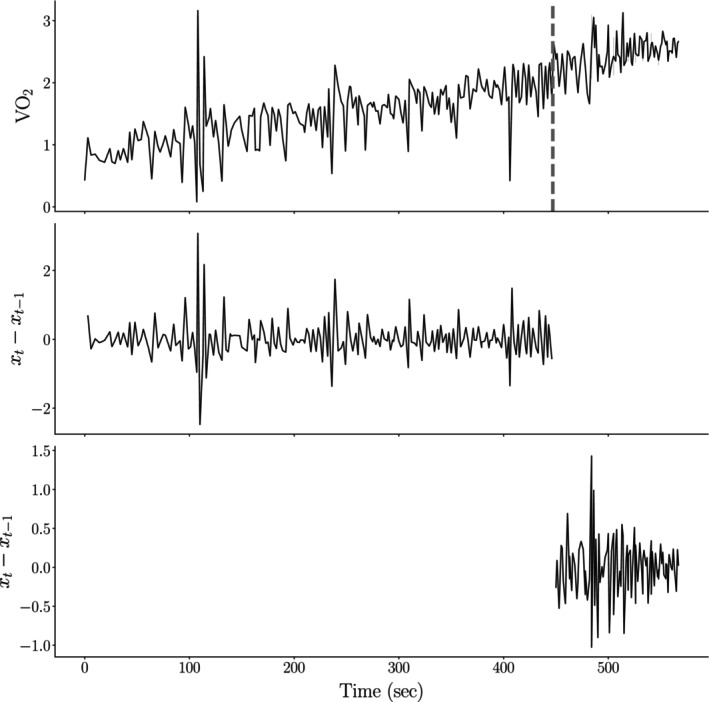
Example of the proposed signal non‐stationarity correction scheme applied to the CPET signal set. The top figure displays the initial V̇O_2_ signal for a participant in our study, with the gray dashed line representing the algorithmically determined VT_1_. Initially, the signal exhibits trend non‐stationarity, evidenced by an Augmented Dickey‐Fuller (ADF) test *p*‐value of p=0.64 (at α=0.05). Application of a differencing scheme both before and after VT_1_ renders both segments of the V̇O_2_ signal statistically weakly stationary ($p\approx 2.46\times 10^{-15}$ and $p\approx 1.30\times 10^{-10}$) for segments before and after VT_1_, respectively, at α=0.05.

Figure 1 provides a visual demonstration of how we difference a CPET signal before and after the detected VT_1_ and evaluate whether it is statistically weakly stationary.

### 2.4 Bayesian statistical testing procedures

In this study, we employed a Bayesian hierarchical model to analyze differences in gas exchange and HR entropy response across sexes, pubertal statuses, and VT_1_ levels. Advancements in Bayesian inference algorithms and software frameworks have improved the accessibility and efficiency of Bayesian techniques (Abril‐Pla et al., 2023; Hoffman & Gelman, 2014).

To define the hierarchical model, we introduce the following notation and sets:

- T represents the set $\left\{\text{Before},\text{After}\right\}$, which indicates whether a signal is observed before or after an individual's VT_1_.
- S denotes the set $\left\{\text{Male},\text{Female}\right\}$.
- P is the set of pubertal statuses: early‐pubertal and late‐pubertal.
- M is the set of CPET metrics for which we computed the SampEn. Specifically, $M=\left\{\dot{V}O_{2},\dot{V}CO_{2},\dot{V}E,\mathrm{RR},\mathrm{HR}\right\}$.
- $I\left(m,p,s,t\right)$ defines the set of indices corresponding to participants with a specific VT_1_ status t, sex s, and pubertal status p, with the CPET metric m.

The model had the following specification:

$$\mu_{p,s,t}^{\left(m\right)}∼N\left(2,1\right)$$

$$\;\sigma_{p,s,t}^{\left(m\right)}∼U\left(0.05,0.50\right)$$

$$\;\nu ∼\log N\left(1,1\right)$$

$$\;y_{i,p,s,t}^{\left(m\right)}∼t\left(\mu_{p,s,t}^{\left(m\right)},\sigma_{p,s,t}^{\left(m\right)},\nu \right)$$

The choice of Gaussian and uniform distributions for the mean and variance priors, respectively, reflects the reasonably bounded range of healthy children's entropy. For the variance prior on μ, we used a larger variance of one to account for the differences between gas exchange entropy and HR entropy.

The prior of $\log N\left(1,1\right)$ for ν reflects our belief that there may be entropy outliers present in the sample, justifying the use of a heavier‐tailed distribution. In a Student‐T distribution, the tails become lighter as ν increases, and when ν>30, the Student‐T distribution approximates a Gaussian distribution (Kruschke, 2013). The $\log N\left(1,1\right)$ prior concentrates approximately 99% of the effective prior probability mass within $\nu \in \left[0,25\right]$ (Lee, 2022).

We employed Markov chain Monte Carlo (MCMC) sampling using the No U‐Turn Sampler algorithm implemented in PyMC (Abril‐Pla et al., 2023), and evaluated convergence using trace plots, effective sample size, and the Gelman‐Rubin R‐hat statistic (Gelman & Rubin, 1992).

In the specified model and subsequent experiments, we will perform multiple comparisons across sex, pubertal status, and work‐rate intensity. Unlike classical methods, a Bayesian hierarchical model inherently addresses the issue of multiple comparisons through its “shrinkage” or partial‐pooling mechanism (Gelman et al., 2012). This approach makes the comparisons more conservative while maintaining appropriate statistical power. Additionally, for each comparison, we will fix two of the levels (e.g., holding sex and pubertal status constant while examining the effect of work rate intensity) to clearly isolate the impact of each factor.

### 2.5 Data description

The protocol was approved by the UC Irvine Institutional Review Board and all methods were performed in accordance with the relevant guidelines and regulations. Written informed consent was obtained from legally authorized guardians and, where appropriate, assent from the participants themselves. We recruited ninety‐five 7‐ to 18‐year‐old participants without any known respiratory, cardiac, or metabolic disease and not taking any chronic prescribed medication (Table 1). We included participants with body mass index (BMI) considered to be within the 95% confidence intervals of calculated age‐dependent BMI percentile. Each volunteer visited the laboratory on six occasions. During the first visit, informed consent was obtained, and demographic and anthropometric data were recorded using a standard calibrated scale and stadiometers, Tanner stage (by questionnaire; height, skin changes, pubic hair and, for males: facial hair and voice changes, and for females: breast development and menses) was assessed and used to determine pubertal status as early pubertal (Tanner 1–2) and late pubertal (Tanner 4–5). Individuals with Tanner 3 were excluded to better distinguish between early‐ and late‐pubertal participants. Furthermore, participants with unstable signals or participants not reaching peak exercise (respiratory exchange rate (RER) >1.0 and/or HR >185) were excluded (Rowland et al., 2008). Table 1 presents relevant summary statistics, including demographic information and clinical measures, for the *n* = 81 male and female (48% male; 52% female) study participants included in the study.

**TABLE 1:** Demographic summary statistics for early‐pubertal and late‐pubertal study participants.

	Males		Females		Combined
	Early‐pubertal *n* = 20	Late‐pubertal *n* = 19	Early‐pubertal *n* = 17	Late‐pubertal *n* = 25	*n* = 81
Age (years)	10.66 ± 1.84	16.81 ± 1.43	9.03 ± 1.24	15.6 ± 1.78	13.28 ± 3.57
Height (cm)	143.54 ± 12.25	172.79 ± 6.71	131.92 ± 9.09	161.19 ± 5.83	153.42 ± 17.40
Total body mass (kg)	36.71 ± 11.78	63.52 ± 10.34	29.72 ± 7.75	55.04 ± 8.06	47.19 ± 16.23
Lean body mass (kg)	24.94 ± 6.45	47.83 ± 6.68	18.99 ± 3.69	35.51 ± 4.42	32.32 ± 11.84
Body fat (%)	28.49 ± 5.90	21.73 ± 5.22	32.81 ± 5.49	32.67 ± 4.98	29.10 ± 6.91
BMI percentile	45.10 ± 29.88	45.68 ± 25.41	49.82 ± 28.14	55.68 ± 23.79	49.49 ± 26.57
Peak V̇O_2_ (mL/min/kg)	52.30 ± 7.67	54.52 ± 8.82	43.81 ± 7.39	40.22 ± 7.69	47.31 ± 9.85
Peak V̇O_2_ (L/min)	1.88 ± 0.46	3.50 ± 0.70	1.31 ± 0.26	2.23 ± 0.50	2.25 ± 0.92

*Note*: Values reflect sample mean and standard deviation.

CPET was completed on the first visit using a ramp type progressive exercise protocol that has been used extensively in children, adolescents, and young adults in our laboratory (Cooper et al., 2014). The participant pedaled on an electronically braked, servo‐controlled CE (Lode, Groningen, The Netherlands) until they reached the limit of tolerance indicated either by the participants themselves or by the assessment of the experienced laboratory staff and faculty. Gas exchange was measured breath‐by‐breath using the Sensor Medics system (Vmax Encore 229, Yorba Linda, CA). HR was measured as the instantaneous value obtained at the given breath. Participants were vigorously encouraged to continue pedaling during the high‐intensity phases of the test. The participants were instructed and monitored to maintain a pedaling rate during exercise between 65 and 75 RPM, and this has proven to be a successful approach to enhance both consistency and standardization in exercise performance, thereby ensuring reliable and comparable data across participants. Tests meeting the criterion of RER >1.0 were deemed valid for analysis. Peak V̇O_2_ was calculated as the highest 20‐s rolling average in the last minute of exercise.

## 3 RESULTS

In the process of our analysis, we developed “EristroPy,” a Python programming language package designed for entropy analysis of short physiological signals. While the primary findings of this paper focus on physiological insights, interested readers can refer to https://zblanks.github.io/eristropy for details on the tool.

Optimal SampEn parameters, $\left(m,r\right)$, for each CPET metric were selected using the method outlined by Blanks and Brown (2024). Choosing suitable values for SampEn parameters, especially for shorter physiological signals, is challenging (Yentes et al., 2013). The proposed scheme automatically determines reasonable settings, validated across synthetic and established signal benchmarks, ensuring stable SampEn estimates.

### 3.1 VT_1_ entropy differences

We conducted a Bayesian analysis to explore the differences in entropy before and after the algorithmically determined VT_1_ in males and females.

Our primary hypothesis was that participants would exhibit higher entropy before the VT_1_ compared to after. To formalize this, we defined $\mu_{p,s,0}^{\left(m\right)}$ as the inferred mean SampEn for participants of pubertal status p and sex s for CPET metric m before the VT_1_, and $\mu_{p,s,1}^{\left(m\right)}$ as the corresponding value after the VT_1_. The difference between the mean SampEn after versus before the VT_1_ was calculated as:

(6) $$\begin{array}{c} \Delta \mu_{s,p}^{\left(m\right)}=\overset{\text{After}\;{\mathrm{VT}}_{1}}{\overbrace{\mu_{s,p,1}^{\left(m\right)}}}-\overset{\text{Before}\;{\mathrm{VT}}_{1}}{\overbrace{\mu_{s,p,0}^{\left(m\right)}}}. \end{array}$$

**FIGURE 2:**
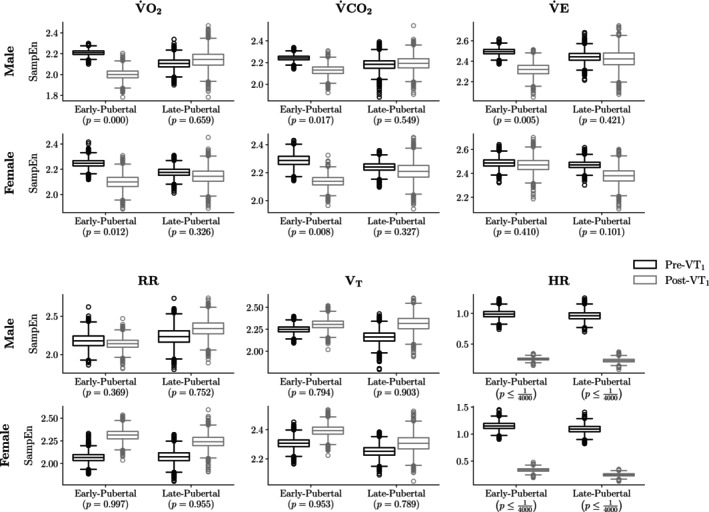
There was a substantial reduction in SampEn for HR and gas exchange metrics (V̇O_2_ and V̇CO_2_) following the VT_1_. Notably, there was a statistically significant 74.9% median decrease in HR entropy across all sex and pubertal status groups. Early‐pubertal males and females exhibited a 5%–10% decline in V̇O_2_ and V̇CO_2_ SampEn post‐VT_1_, aligning with previous findings and our initial hypothesis. However, this trend diverged in late‐pubertal individuals, where females showed smaller decreases and males exhibited no statistically significant change. In contrast, RR SampEn increased after VT_1_. Each box‐and‐whisker plot displays the distribution's quartiles, with the central line indicating the median. The “whiskers” extend to the smallest and largest values within 1.5 interquartile ranges from the lower and upper quartiles, while any observations outside this range are shown as individual points.

Given that the Bayesian model defines a complete probabilistic system, we estimated the probability that $\Delta \mu_{s,p}^{\left(m\right)}$ is greater than zero by calculating:

(7) $$\begin{array}{c} P\left(\Delta \mu_{s,p}^{\left(m\right)}\geq 0|y\right)\approx \frac{1}{D}\sum_{k=1}^{D}I\left[\Delta \mu_{s,p}^{\left(m\right)}\left(k\right)\geq 0\right], \end{array}$$

where D represents the number of posterior draws from the MCMC (4000 in our case), and $I\left[\cdot \right]$ denotes the indicator function. Figure 2 displays the results of this analysis.

From the results presented in Figure 2, we highlight several findings:

- For early‐pubertal males and females, gas exchange metrics such as V̇O_2_ and V̇CO_2_ showed a relative decrease in SampEn of 5%–10% following the VT_1_. The probability that entropy was greater after the VT_1_ than before it, for V̇O_2_ and V̇CO_2_, was approximately 0.01. This observation aligns with previous research and corroborates our initial hypothesis (Blanks, Brown, Cooper, et al., 2024).
- In contrast, late‐pubertal females had directionally consistent SampEn decreases, albeit smaller in magnitude. For late‐pubertal males, their mean Δμ posterior credible interval contained zero, suggesting limited statistical evidence of a relative decrease in V̇O_2_ and V̇CO_2_ after the VT_1_.
- The RR SampEn difference deviated from the general trend, showing an average increase post‐VT_1_ across all sex and pubertal‐status combinations.
- The relative SampEn decrease in HR was more pronounced than other gas exchange metrics. Across different combinations, the median value was −74.9%. Moreover, out D=4000 posterior draws, not a single instance had an HR Δμ greater than or equal to zero.

### 3.2 Breath‐by‐breath record and SampEn estimates

**FIGURE 3:**
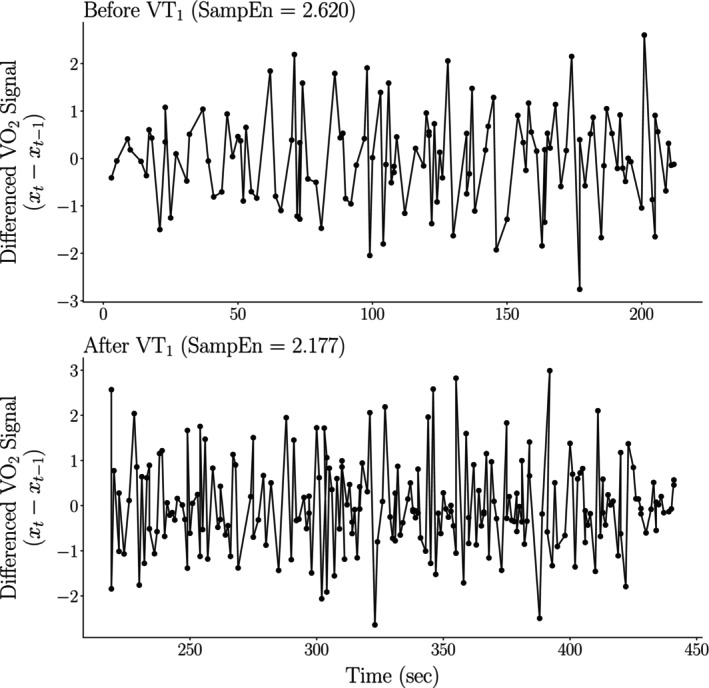
Despite having a larger standard deviation, the SampEn of the breath‐by‐breath V̇O2 record was lower post‐VT_1_ compared to pre‐VT_1_ (2.177 vs. 2.620, respectively).

In Figure 2, we presented the observation that, on average, an individual's SampEn decreased following the VT_1_. To provide greater intuition for this finding and to highlight the relationship between the breath‐by‐breath data and the derived SampEn values, we present an example of the input breath‐by‐breath record and its corresponding SampEn estimates. Figure 3 illustrates the breath‐by‐breath V̇O_2_ data for a representative participant, segmented into periods before and after the VT_1_.

Despite the breath‐by‐breath V̇O_2_ signal having a greater standard deviation post‐VT_1_ (*σ* ≈ 0.463) versus pre‐VT_1_ (*σ* ≈ 0.393), the participant exhibited lower SampEn after their ventilatory threshold. This is because, on average, the signal becomes more predictable, as demonstrated by the oscillating up‐and‐down pattern observed in Figure 3. This finding underscores the important fact that while entropy and variance can be related, they capture different properties of a signal.

### 3.3 HR SampEn percent difference explained

**FIGURE 4:**
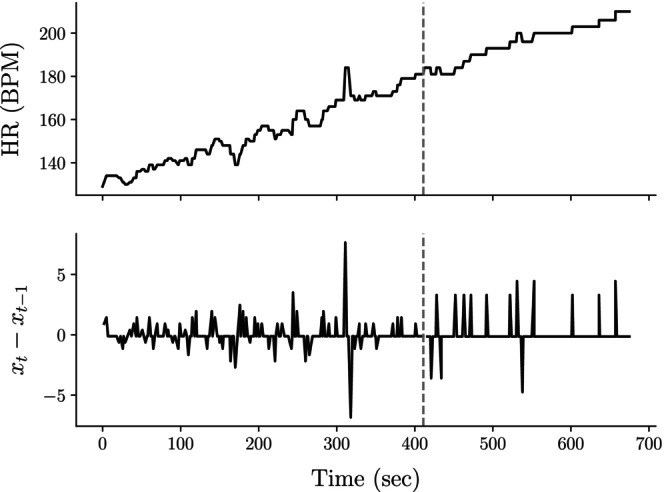
HR becomes markedly more predictable after the VT_1_. This decrease can be attributed to the plateauing of HR signals, as evident in the comparison between the raw and differenced HR signals across the CPET. The top plot shows the overall HR trajectory, while the bottom plot, used for SampEn calculations, highlights the reduced variability and increased predictability beyond the VT_1_ (indicated by the gray dashed line). This plateauing phenomenon is likely due to the autonomic regulation of HR reaching an upper limit at elevated work rates, which we posit as the primary cause for the observed decrease in HR SampEn.

We found that the median decrease in HR SampEn before versus after the VT_1_ was 74.93%. We hypothesize that the significant relative decrease in HR SampEn may be attributed to an individual's HR plateauing after the VT_1_, resulting in highly predictable CPET signals. Figure 4 illustrates this phenomenon.

Before the VT_1_, the HR signal exhibits variability, reflected by spikes and valleys in the differenced HR signal (bottom plot). However, after the VT_1_, the HR often appears to plateau, leading to a highly predictable signal and substantially lower entropy.

Since HR was derived from instantaneous values at each breath, and participants exhibited higher breathing frequencies beyond the VT_1_, the data collection process may have caused artificial smoothing of the HR signal, potentially decreasing its overall entropy. Nevertheless, the substantial entropy reduction observed suggests that even with potential smoothing, the drop‐off in entropy was significant. A more granular analysis of the beat‐to‐beat entropy of HR CPET signals is an area of future investigation.

### 3.4 Between‐sex SampEn differences

Previously, we observed, on average, that female adolescents exhibited relatively greater SampEn than their male counterparts. To test this hypothesis, we evaluated the relative SampEn difference for each CPET metric, VT_1_ state, and pubertal status. Specifically, we evaluated the posterior quantity:

(8) $$\begin{array}{c} \Delta \mu_{p,t}^{\left(m\right)}=\left(\frac{\overset{\text{Male SampEn}}{\overbrace{\mu_{0,p,t}^{\left(m\right)}}}-\overset{\text{Female SampEn}}{\overbrace{\mu_{1,p,t}^{\left(m\right)}}}}{\mu_{1,p,t}^{\left(m\right)}}\right)\times 100, \end{array}$$

where $\mu_{0,p,t}^{\left(m\right)}$ represents the mean posterior SampEn estimate for CPET metric m for male participants of pubertal status p and VT_1_ status t. Conversely, $\mu_{1,p,t}^{\left(m\right)}$ corresponds to the same measure for female participants. Unlike the quantity calculated in above equation (Pincus et al., 2000), $\Delta \mu_{p,t}^{\left(m\right)}$ represents the *percent* difference in SampEn between male and female participants for CPET metric m, pubertal status p, and VT_1_ state t. A negative value for $\Delta \mu_{p,t}^{\left(m\right)}$ indicates, on average, lower SampEn in males compared to females. The results of this analysis are presented in Table 2.

**TABLE 2:** The values for $\Delta \mu_{p,t}^{\left(m\right)}$ represent the mean ± standard deviation of the percent difference between male and female participants as calculated in equation (Solís‐Montufar et al., 2020).

	Early‐pubertal		Late‐pubertal	
	$\Delta \mu_{p,t}^{\left(m\right)}$ (percent difference)	$P\left(\Delta \mu_{p,t}^{\left(m\right)}\geq 0|y\right)$	$\Delta \mu_{p,t}^{\left(m\right)}$ (percent difference)	$P\left(\Delta \mu_{p,t}^{\left(m\right)}\geq 0|y\right)$
Before VT_1_				
V̇O_2_	−1.20 ± 1.83	0.25	−3.06 ± 2.80	0.13
V̇CO_2_	−2.38 ± 2.15	0.13	−2.26 ± 2.90	0.21
V̇E	0.71 ± 1.85	0.65	−1.52 ± 2.52	0.26
RR	−2.83 ± 3.83	0.23	−5.04 ± 5.82	0.18
V_T_	−2.51 ± 2.48	0.15	−4.69 ± 3.82	0.10^†^
HR	−13.9 ± 7.3	0.04^*^	−11.5 ± 9.1	0.11
After VT_1_				
V̇O_2_	−4.66 ± 3.55	0.10^†^	−0.09 ± 4.79	0.49
V̇CO_2_	−0.41 ± 2.83	0.44	−1.14 ± 3.96	0.38
V̇E	−6.20 ± 3.93	0.06^†^	1.73 ± 5.17	0.64
RR	−3.65 ± 3.99	0.17	9.38 ± 6.11	0.94
V_T_	−4.31 ± 2.85	0.07^†^	−0.08 ± 4.84	0.49
HR	−22.9 ± 10.4	0.02^*^	−5.2 ± 18.2	0.34

* *p* < 0.05.

† *p* < 0.1; *p* > 0.05.

There is a consistent trend where females generally exhibit higher relative SampEn compared to males, with this contrast most pronounced in HR SampEn differences among early‐pubertal participants, supporting prior findings of sex‐related disparities in SampEn. Across both early‐ and late‐pubertal phases, males, on average, exhibited lower SampEn than females particularly for metrics such as HR. However, it is important to note that many observed differences lack statistical significance, as indicated by the probability values for $\Delta \mu_{p,t}^{\left(m\right)}$ not falling below the 0.05 threshold.

### 3.5 Pubertal SampEn differences

To examine entropy disparities between early‐ and late‐pubertal children, we analyzed SampEn across the entire CPET signal without partitioning at the VT_1_. We applied the differencing procedure detailed in Equation (Kiefer et al., 2021) to generate weakly stationary signals and determined optimal SampEn parameters using the methodology outlined by Blanks and Brown (2024).

The Bayesian hierarchical model employed to assess SampEn variations between early‐ and late‐pubertal children resembles the one previously detailed, but without a “plate” for the set T due to not partitioning at the VT_1_. We hypothesized that late‐pubertal children would exhibit lower SampEn values compared to their early‐pubertal counterparts.

Applying this Bayesian model without segmenting at the VT_1_, we calculated the average mean posterior SampEn percentage difference among participants of a given sex s as follows:

(9) $$\begin{array}{c} \Delta \mu_{s}^{\left(m\right)}=\left(\frac{\overset{\text{Late}-\text{Pubertal SampEn}}{\overbrace{\mu_{s,1}^{\left(m\right)}}}-\overset{\text{Early}-\text{Pubertal SampEn}}{\overbrace{\mu_{s,0}^{\left(m\right)}}}}{\mu_{s,1}^{\left(m\right)}}\right)\times 100.\; \end{array}$$

**FIGURE 5:**
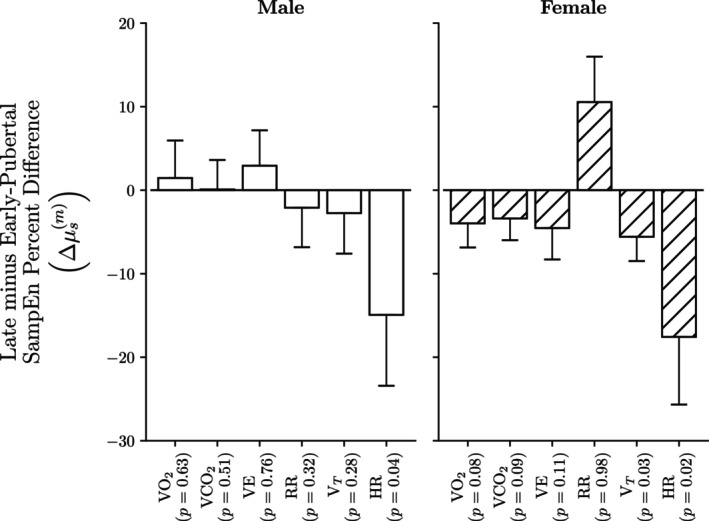
There is a statistically significant 15%–20% decrease in HR SampEn between late versus early‐pubertal participants for both males and females. Although late‐pubertal children generally exhibited lower mean gas‐exchange SampEn, these differences did not reach statistical significance. Each bar plot displays the mean posterior percent difference between late‐ and early‐pubertal participants for each (metric, sex) combination, with error bars representing one standard deviation of the posterior variance.

The outcomes of this analysis are presented in Figure 5.

### 3.6 HR SampEn comparison

Finally, to compare the overall SampEn relative difference between HR and the other gas exchange metrics, we evaluated the SampEn of HR for a given sex, pubertal, and VT_1_ status combination versus the minima of the gas exchange metrics for that same combination. That is:

(10) $$\begin{array}{c} \tilde{\Delta \mu_{s,p,t}}=\left(\frac{\overset{\mathrm{HR}\;\text{SampEn}}{\overbrace{\mu_{s,p,t}^{\left(\mathrm{HR}\right)}}}-\overset{\mathrm{Min}\;\mathrm{Gas}\;\text{Exchange SampEn}}{\overbrace{\mu_{s,p,t}^{\left(\neg \mathrm{HR}\right)}}}}{\mu_{s,p,t}^{\left(\neg \mathrm{HR}\right)}}\right)\times 100. \end{array}$$

In above equation (Pincus et al., 2000), $\mu_{s,p,t}^{\left(\mathrm{HR}\right)}$ represents the mean posterior SampEn estimate of HR for participants of sex s, pubertal status p, and VT_1_ status t. The quantity, $\mu_{s,p,t}^{\left(\neg \mathrm{HR}\right)}$, is the minimum gas exchange SampEn value for the same combination. Table 3 displays the results of this comparison.

**TABLE 3:** For all combinations, the HR SampEn is significantly lower than the corresponding gas exchange metric, and this difference increases going from before to after the V_T_.

	Early‐pubertal		Late‐pubertal	
	$\tilde{\Delta \mu_{s,p,t}}$ (percent difference)	$P\left(\tilde{\Delta \mu_{s,p,t}}\geq 0|y\right)$	$\tilde{\Delta \mu_{s,p,t}}$ (percent difference)	$P\left(\tilde{\Delta \mu_{s,p,t}}\geq 0|y\right)$
Males				
Before VT_1_	−49.5 ± 3.6	$\leq {\frac{1}{4000}}^{**}$	−49.6 ± 4.5	$\leq {\frac{1}{4000}}^{**}$
After VT_1_	−86.6 ± 1.3	$\leq {\frac{1}{4000}}^{**}$	−88.5 ± 1.7	$\leq {\frac{1}{4000}}^{**}$
Females				
Before VT_1_	−42.8 ± 3.5	$\leq {\frac{1}{4000}}^{**}$	−46.0 ± 3.8	$\leq {\frac{1}{4000}}^{**}$
After VT_1_	−83.5 ± 1.7	$\leq {\frac{1}{4000}}^{**}$	−87.5 ± 1.6	$\leq {\frac{1}{4000}}^{**}$

*Note*: The values for ${\tilde{\Delta \mu }}_{s,p,t}$ correspond to the mean ± standard deviation percent difference between HR SampEn and the minimum gas exchange SampEn value for the same combination.

** *p* < 0.01.

## 4 DISCUSSION

### 4.1 Approach to SampEn in nonstationary, short duration progressive CPET

Our data suggest that even with the constraints of standardized CPET progressive exercise testing, signal analytics such as SampEn are technically feasible. As shown in Figures 2 and 5 and Tables 2 and 3, we demonstrated both effective informatics methods and new insights into gas exchange and HR responses to exercise in light of maturational, sex, and work intensity factors. Within our study, we introduced EristroPy, a Python package designed to facilitate the application of entropy analysis to CPET and time‐series signals. EristroPy played a pivotal role in ensuring the validity of our entropy estimates by addressing key technical aspects commonly occurring during a CPET: signal non‐stationarity and shorter signal durations.

- *Signal stationarity*: A fundamental requirement for reliable entropy calculations is the assumption of weak stationarity (Chatain et al., 2020). Due to the increasing work rate throughout the CPET, it is quite probable that the input signals will be non‐stationary because of an increasing trend throughout the test. EristroPy automatically detects and corrects this issue for all input signals laying the foundation for reliable entropy analysis.
- *Entropy parameter selection and uncertainty quantification*: The technical process by which EristroPy automatically selects SampEn parameters, while important, is beyond the scope of this paper (see Blanks and Brown for more details (Blanks & Brown, 2024)). Inappropriate SampEn parameters can yield unstable or noninformative estimates leading to meaningless conclusions (Pincus & Goldberger, 1994). This challenge is amplified when dealing with shorter signal lengths typically obtained from a CPET. Most biomedical entropy analyses assume one has access to signals with multiple hundreds to thousands of observations; substantially less work has been done using shorter signals, and the guidance provided to researchers has been limited (Yentes et al., 2013). Under the EristroPy framework, we implicitly account for the issue of shorter signals by automatically selecting optimal SampEn parameters under this constraint and quantify the corresponding uncertainty of the SampEn estimate. Moreover, we provide EristroPy as an open‐source tool accessible to the wider clinical and research community. We hope that by developing this framework, it will allow greater accessibility and reproducibility within the community.

### 4.2 The challenge of entropy analysis for averaged CPET signals

This study involved analyzing breath‐by‐breath signals before and after an individual's VT_1_, typically containing 100–200 observations. While the EristroPy framework enabled reliable SampEn estimation for such inputs, the standard practice of averaging CPET signals over an 8–12 min protocol (Takken et al., 2009), often yields signal sets with fewer than 50 observations. This averaging operation poses two main challenges for entropy analysis.

First, signal averaging is inherently “lossy,” altering the signal's shape, amplitude, and reducing total information (Kawala‐Sterniuk et al., 2020). Consequently, entropy computed on averaged signals may yield lower values compared to those calculated on non‐smoothed signals.

Second, research indicates that SampEn estimates are generally unreliable for signals with fewer than 50 observations due to insufficient template matches, leading to undefined SampEn values (Li et al., 2015).

In scenarios where entropy analysis on averaged CPET signal sets is desired, alternative entropy metrics such as permutation entropy (PermEn) may be considered (Bandt & Pompe, 2002). Recent studies have demonstrated that a Bayesian reformulation of PermEn allows for reliable entropy estimates even with as few as 10 observations (Blanks, Brown, Adams, & Angadi, 2024). However, the development of a corresponding Bayesian reformulation for SampEn remains a topic for future investigation.

### 4.3 Diagnostic and research use of SampEn quantification in pediatric CPET

Using the analysis described above to measure SampEn in progressive exercise CPET in early and late pubertal children and adolescents, we were able to identify a number of insights into physiological responses to exercise including: (1) existence of developmental stage differences in physiological responses to exercise; in particular, the reduction in SampEn in the early pubertal participants as the ramp work intensity increased (Figure 2); (2) effects related to biological sex (Table 2 and Figure 5); and (3) markedly lower entropy in HR compared with the gas exchange variables (V̇O_2_, V̇CO_2_, V̇E, RR, and V_T_)—Table 3.

In the early‐pubertal participants, but not the late‐pubertal participants, we found a significant reduction in SampEn in key gas exchange variables during CPET in the >V_T_ phase of the ramp CPET (Figure 2). This was observed from only several minutes of data in the ramp protocol, but, nonetheless, corroborates our recent findings using a 30‐min protocol, the MBEB (Blanks, Brown, Cooper, et al., 2024). When considering the combined SampEn for the whole ramp, we also found evidence of maturational differences, most notably in the HR response. Consistent with our previous observations in the MBEB protocols, HR SampEn was significantly higher in the early compared with late pubertal participants (Figure 5).

There are few systematic studies of maturational effects on SampEn physiological responses to exercise. Wu et al. (2016) analyzed stride patterns and found general reductions in SampEn as children aged from 3 to 14 years‐old. Kiefer et al. (2021) found that sample entropy analysis of postural control mechanisms decreased from late‐childhood to mid‐adolescence. Earlier, pioneering studies of entropy in hormonal regulation demonstrated that approximate entropy of pituitary growth hormone secretion patterns declined dramatically in males as they transitioned to late puberty (Pincus et al., 2000). We speculate that the observed pubertal status dependent reduction in several physiological variables associated with acute exercise may reflect overall maturation of physiological control as children transition to adolescence and young adulthood.

Little is currently known about the response of respiratory (gas exchange) signal transduction to work intensity, but there is evidence that SampEn of respiratory variables is complex and dependent on intensity and duration of the exercise input (Lai et al., 2023). There are extensive analyses of HR variability and a variety of information‐entropy methodologies during exercise. These studies in general demonstrate that HR signal entropy lessens as the exercise work intensity increases (Berry et al., 2022; Fukumoto et al., 2022; Solís‐Montufar et al., 2020).

While the mechanism of the reduction in SampEn with increasing work intensity is not entirely clear, we speculate that as physiological systems adjust to perturbations of increasing magnitude, system control becomes more effective as competing regulatory inputs are diminished and SampEn is reduced. An explanation for the significant reduction in SampEn with exercise intensity that we observed only in the early pubertal participants might be related to the younger subjects generally higher levels of physiological entropy and the subsequent modulating effect of increasing work intensity as the ramp protocol proceeded.

In this context, one must differentiate between the behavior of physiological system entropy in the acute response to a perturbation like exercise with the changes that might occur in physiological entropy in the baseline state with reference to global physical fitness or in response to formal exercise training. Although there are limited data addressing the relationship between physiological entropy at baseline and CRMF, in an earlier study, Soares‐Miranda et al. (2011) found that HR SampEn at rest was greater in young adults whose habitual levels of physical activity were estimated to be vigorous. An intriguing possibility, one awaiting future studies, is that one effect of increased physical fitness is greater complexity in baseline levels of physiological control perhaps benefitting adjustment to environmental perturbations, like exercise, to cellular homeostasis.

As shown in Figure 2, HR SampEn was much lower than the other gas exchange CPET variables and was more robustly reduced when the work rate exceeded the V_T_. To the best of our knowledge, there are few studies that have directly compared SampEn of HR and gas exchange variables during exercise. HR signal entropy appears to reflect predominantly autonomic control, namely, a balance of sympathetic and parasympathetic activity (Fukumoto et al., 2022). In contrast to HR, CPET gas exchange variables that are more dependent on RR and tidal volume (V_T_) are influenced by cognitive control processes (e.g., the ability to alter respiration while speaking, etc.) (Grassmann et al., 2016). The contribution of these additional respiratory control mechanisms may explain the much greater SampEn compared to HR observed in our study.

Consistent with our previous MBEB study, males tended to have subtle but lower overall SampEn than females in several CPET variables. As noted, in the early pubertal males, V̇O_2_ and V_T_ were about 5% and 6%, respectively, lower than females. In the late pubertal males, RR rate was about 13% lower than females.Kuo and Yang. (2002) studied approximate entropy of HR signals in middle‐aged men and women and also found greater entropy in the female participants. This sexual dimorphism in the stress response might be attributed to the notion, as suggested earlier by Taylor et al. (2000) that while males experience the conventional *fight‐or‐flight* response to stressors, females exhibit a *tend‐and‐befriend* response in which they employ social coping methods to combat stress; the latter, one might speculate, involving a more complex physiological control paradigm.

Just as HRV is increasingly used clinically to assess clinical conditions such as heart failure, signal transduction parameters such as SampEn, may become clinically useful in a variety of diseases. The fact that entropy is reduced at higher work rates when metabolic demand is increased, strongly suggests that entropy may be useful under disease conditions when the normal adaptive physiological mechanisms are limited. This will be determined in future research focused on disease.

## 5 CONCLUSIONS

Patterns of habitual physical activity (HPA) in children are intuitively described as complex and disordered. Direct observation (Bailey et al., 1995; Berman et al., 1998), HR monitoring (Gilliam et al., 1981), and more modern wearable, actigraphy investigations employing neural network and Artificial intelligence/Machine learning (AI/ML) approaches (An et al., 2024; Ellingson et al., 2016; Freedson et al., 2011) support this notion and characterize HPA in children and adolescents as consisting of intermittent brief bouts of exercise and rest of varying intensity and duration. In a recent study, we demonstrated the effect of maturational status, work intensity, and biological sex on SampEn during MBEB protocols developed by our group specifically to more closely mimic naturalistic patterns of exercise in youth (Blanks, Brown, Cooper, et al., 2024). The most salient maturational CPET differences as children mature are arguably the large differences in the kinetics of V̇CO_2_ and associated ventilation and HR, the responses of which are considerably faster in early pubertal children (Armon et al., 1991; Baraldi et al., 1991; Cooper et al., 1987). Younger children demonstrate great amounts of oxygen for work performed (Zanconato et al., 1991), possibly indicating a less well‐developed metabolic machinery for anaerobic energy production. Whether the previously demonstrated maturational aspects of the physiological response to exercise are mechanistically related to signal control factors such as entropy remains to be determined.

Additionally, conventional CPET is increasingly used as a diagnostic and monitoring tool for a wide range of pediatric conditions and diseases (Cooper et al., 2022). The standard progressive work rate CPET protocols do not mimic the start‐stop pattern of exercise, but standard CPET does elicit physiologic responses across a wide range of work intensities over a relatively short interval (ideally, 10–12 min). Accordingly, summarizing the response to ramp CPET via a parsimonious yet statistically complex estimator via SampEn may prove to have effective clinical and research applications in better understanding physiological responses to exercise in children and adolescents in health and disease.

## AUTHOR CONTRIBUTIONS

*Conceived and designed research*: Dan M. Cooper, Ronen Bar‐Yoseph, Shlomit Radom Aizik; *Performed experiments*: Zachary Blanks; *Analyzed data*: Zachary Blanks; *Interpreted results of experiments*: Zachary Blanks, Donald E. Brown, Dan M. Cooper; *Prepared figures*: Zachary Blanks, Dan M. Cooper; *Drafted manuscript*: Zachary Blanks, Dan M. Cooper; *Edited and revised manuscript*: Dan M. Cooper, Donald E. Brown, Ronen Bar‐Yoseph, Shlomit Radom Aizik; *Approved final version of manuscript*: Dan M. Cooper.

## FUNDING INFORMATION

The work reported in this paper was partially supported by a grant, Award UL1TR003015, from the National Center for Advancing Translational Science of the National Institutes of Health. It was also supported by UC Irvine CTSA UL1TR001414 and the Pediatric Exercise and Genomics Research Center Project REACH U01TR002004.

## CONFLICT OF INTEREST STATEMENT

The authors declare no conflicts of interest.

## ETHICS STATEMENT

All human participant procedures performed in this study were approved by the Institutional Review Board of the University of California at Irvine and appropriate informed consent and assent were obtained.

## Data Availability

The data used in this study is available upon reasonable request to the corresponding author.
